# Inadequate Triglyceride Management Worsens the Durability of Dipeptidyl Peptidase-4 Inhibitor in Subjects with Type 2 Diabetes Mellitus

**DOI:** 10.1155/2017/5856475

**Published:** 2017-05-25

**Authors:** Masashi Shimoda, Maiko Miyoshi-Takai, Shintaro Irie, Akihito Tanabe, Atsushi Obata, Seizo Okauchi, Hidenori Hirukawa, Tomohiko Kimura, Kenji Kohara, Shinji Kamei, Tomoatsu Mune, Kohei Kaku, Hideaki Kaneto

**Affiliations:** ^1^Division of Diabetes, Metabolism and Endocrinology, Kawasaki Medical School, 577 Matsushima, Kurashiki 701-0192, Japan; ^2^Division of General Internal Medicine 1, Kawasaki Medical School, 577 Matsushima, Kurashiki 701-0192, Japan

## Abstract

Dipeptidyl peptidase-4 (DPP-4) inhibitors are often used all over the world and exert various beneficial effects including glucose-lowering effect in many subjects with type 2 diabetes. It is poorly understood, however, which factors are closely related with the durability of glucose-lowering effect by DPP-4 inhibitor. In this study, we examined retrospectively which factors could mainly influence the durability of DPP-4 inhibitor. We enrolled 212 participants with type 2 diabetes to whom DPP-4 inhibitor was administered for over 1 year without an addition or increase of other hypoglycemic agents. Age and baseline HbA1c level were significantly higher in the effective group than those in the ineffective group. The effective group had a tendency of smaller amounts of weight change, average total cholesterol, and average triglyceride compared with the ineffective group. Multiple logistic regression analysis showed that average triglyceride and baseline HbA1c were independent predictors associated with the durability of DPP-4 inhibitor. Moreover, an average triglyceride level contributed to the durability of DPP-4 inhibitor in the obese group (BMI ≥ 25 kg/m^2^) but not in the nonobese group (BMI < 25 kg/m^2^). These results suggest the importance of strict triglyceride management to maintain the durability of glucose-lowering effect by DPP-4 inhibitor, especially in obese subjects with type 2 diabetes.

## 1. Introduction

The pathophysiology of type 2 diabetes is characterized by relative insulin secretory failure against insulin resistance. It is widely accepted that *β*-cell function progressively deteriorates in individuals with type 2 diabetes mellitus as previously shown in the UK Prospective Diabetes Study [1]. There is some variation in pancreatic *β*-cell response to various stimuli (e.g., insulin resistance, increased metabolic load, and systemic inflammation) among individuals, not only due to the difference of genetic susceptibility but also due to environmental stress such as inflammatory stress, endoplasmic reticulum stress, metabolic and oxidative stress (e.g., glucotoxicity, lipotoxicity, and glucolipotoxicity), amyloid stress, and loss of islet cell integrity [2].

The incretin hormones, glucagon-like peptide-1 (GLP-1) and glucose-dependent insulinotropic polypeptide (GIP), are secreted from the gastrointestinal tract along with the digestion and absorption of nutrients and promote glucose-dependent insulin secretion from pancreatic *β*-cells [3, 4]. Although incretin secretion from gastrointestinal tract is not necessarily decreased in subjects with type 2 diabetes, it is likely that impaired incretin effect is another characteristic of type 2 diabetes [5–8]. It has been thought that the reduced incretin effect in type 2 diabetes is due to some defects in incretin receptor signaling rather than the decrease of blood concentration of incretin hormones. Previous reports showed that the expression levels of incretin receptor were decreased with hyperglycemia which likely leads to the impaired incretin effects found in diabetes [8, 9]. Furthermore, the reduction of glucotoxicity with some glucose-lowering drugs restored the expression of incretin receptors in diabetic animals [9, 10] and improved incretin-induced insulin secretion in subjects with type 2 diabetes [11]. These data suggest that the durability of incretin-based therapy is likely dependent on the condition of glycemic control.

Alteration of lipid metabolism is one of the important characteristics that link the relationship between obesity and type 2 diabetes [12–15]. Abnormal lipid metabolism affects both insulin resistance and pancreatic *β*-cell function [15–17]. For example, prolonged exposure to fatty acid reduces pancreatic *β*-cell function and mass (i.e., lipotoxicity) [15, 18], whereas short-term exposure facilitates insulin secretion [15, 19]. Recently, it was reported that while incretin secretion was comparable between obese and nonobese subjects with type 2 diabetes [20], glucose tolerance and obesity impaired the incretin effect independently of other factors [21]. Furthermore, Knop et al. showed that the incretin action was deteriorated even in obese participants who have normal glucose tolerance in spite of their insulin-resistant state and that the coexistence of obesity and hyperglycemia resulted in the additive attenuation of the incretin effect [22]. About the relationship between lipid control and incretin action, Kang et al. reported that elevated fatty acid contributes to impaired responsiveness to GLP-1, partially through the downregulation of GLP-1 receptor signaling and the improvement of lipid control by using fibrate in mouse models of obesity and diabetes [23]. Hodson et al. also showed that lipotoxicity disrupted incretin-regulated human *β*-cell connectivity, whereas GLP-1 and GIP recruit a robust coordinated subnetwork of *β*-cells [24]. Taken together, lipotoxicity is likely involved in the impaired response to incretin in pancreatic *β*-cells.

Dipeptidyl peptidase-4 (DPP-4) inhibitor suppresses activity of DPP-4, which is a splitting enzyme of incretin, and thereby increases serum levels of GLP-1 and GIP. Both incretins stimulate insulin secretion from *β*-cells in a glucose-dependent manner, and GLP-1 suppresses glucagon secretion from *α*-cells. At present, DPP-4 inhibitor is very often used for the treatment of type 2 diabetes mellitus and exerts beneficial effects including glucose-lowering effect in many subjects with type 2 diabetes. Especially, DPP-4 inhibitor is widely used in Japan, because the pathophysiology of type 2 diabetes in Japanese is primarily characterized by *β*-cell dysfunction and less insulin resistance compared with that in Caucasians [25]. However, to our knowledge, although previous reports have suggested the importance of weight management and diet/exercise therapy as the predictors for the durability of DPP-4 inhibitor [26, 27], it remains unclear which factors are closely associated with the durability of glucose-lowering effect by DPP-4 inhibitor. In this study, to examine which factors could influence the durability of DPP-4 inhibitor in subjects with type 2 diabetes, we analyzed retrospectively 212 outpatients who were treated with DPP-4 inhibitor for over 12 months without an addition or increase of other antidiabetes agents.

## 2. Materials and Methods

### 2.1. Subjects

The participants with type 2 diabetes who visited the outpatient clinic of diabetes at Kawasaki Medical School Hospital and had dipeptidyl peptidase-4 (DPP-4) inhibitors, that is, sitagliptin 50 mg/day, vildagliptin 100 mg/day, and alogliptin 25 mg/day, for over 1 year were eligible in this study. In addition, we excluded the subjects with the following states: (1) addition of other hypoglycemic agents, (2) increase of other glucose-lowering agents, (3) using steroid and/or immunosuppressive agents, and (4) suffering malignancy, acute and chronic inflammatory disease, and endocrine disease. The hospital ethics committee approved the study protocol (numbers 1183 and 1183-1), and informed consent was obtained from each person by showing it in our homepage after a full explanation of the study.

### 2.2. Examination Items

We monitored various clinical parameters such as HbA1c, body weight, total cholesterol, triglyceride, HDL cholesterol, and LDL cholesterol at baseline, 2, 4, 6, 8, 10, and 12 months after administrating DPP-4 inhibitors. Blood samples were collected at various time points when the participants visited the hospital. To elucidate the factors associated with the efficacy of DPP-4 inhibitor in participants with type 2 diabetes, we divided the subjects into the effective group and the ineffective group and compared the participant backgrounds of the effective group with those of the ineffective group. It is noted here that effectiveness was defined as follows: when there was no change or decrease of HbA1c level from baseline to 12 months in case of baseline HbA1c < 7% (53 mmol/mol) or when there was a decrease of HbA1c level from baseline to 12 months in case of baseline HbA1c ≥ 7% (53 mmol/mol).

### 2.3. Statistical Analyses

All analyses were performed by using JMP version 9. The comparison of clinical characteristics between the effective group and ineffective group was analyzed by employing the Mann–Whitney *U* analysis and *χ*^2^ test. The Kruskal-Wallis test and Steel-Dwass test were used for comparisons of the values of HbA1c between baseline and after treatment. Multiple logistic regression method was used to analyze the factors independently contributing to the efficacy of DPP-4 inhibitor in participants with type 2 diabetes.

## 3. Results

### 3.1. Characteristics of the Study Participants

A total of 212 participants (men 126, 59%; women 86, 41%) were enrolled in this study. The baseline characteristics of the study participants were as follows: age 64.3 ± 12.0 years; body weight 65.4 ± 15.8 kg; BMI 25.1 ± 5.1 kg/m^2^; duration of diabetes 15.1 ± 9.2 years; HbA1c 7.7 ± 1.2% (61 ± 13.2 mmol/mol); total cholesterol 182 ± 34 mg/dl; triglyceride 128 ± 80 mg/dl; HDL cholesterol,55 ± 14 mg/dl; and LDL cholesterol 99 ± 27 mg/dl. In addition, the prevalence rates of neuropathy, nephropathy, and retinopathy were 54%, 28%, and 35%, respectively. Therapeutic situation before the administration of DPP-4 inhibitor was as follows: diet only 15%; sulfonylurea 35%; glinide 15%; biguanide 42%; thiazolidine 39%; a-glucosidase inhibitor 26%; insulin 15%; monotherapy 26%; and combination therapy 58% (2 kinds of medicine 32%; 3 kinds 22%; and 4 kinds 5%).

### 3.2. Glycemic Control after Administrating DPP-4 Inhibitors

**Figure 1:**
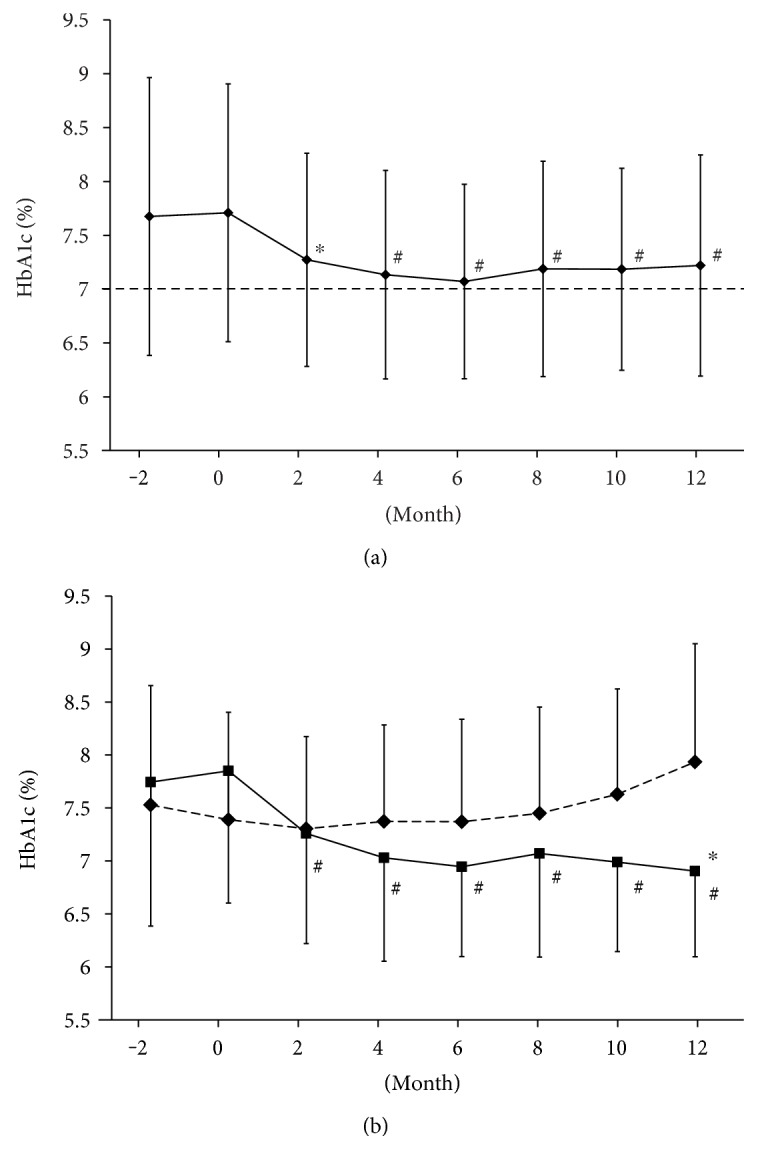
(a) Alteration of HbA1c levels after starting DPP-4 inhibitor in all participants (overall trend: *p* < 0.0001). ^∗^*p* < 0.0005 versus baseline; ^#^*p* < 0.0001 versus baseline. Data are shown as mean ± SD. (b) Alteration of HbA1c levels after starting DPP-4 inhibitor in the effective group (solid line) (overall trend: *p* < 0.0001) and the ineffective group (dashed line) (overall trend: *p* < 0.01). ^∗^*p* < 0.0001 versus the ineffective group at the same time point; ^#^*p* < 0.0001 versus baseline in the effective group. Data are shown as mean ± SD.

DPP-4 inhibitors significantly lowered HbA1c levels 2 months after starting them compared to baseline in all participants in this study (Figure 1(a)). The change of HbA1c from baseline to 6 months later was −0.7 ± 1.1% (7.7 ± 12.0 mmol/mol). HbA1c level was maintained even after reaching trough 6 months after starting the therapy (Figure 1(a)), and the change of HbA1c from baseline to 12 months later was −0.5 ± 1.1% (5.5 ± 12.0 mmol/mol).

### 3.3. Difference of Clinical Background between the Effective Group and Ineffective Group

There seemed to be a large variation among subjects in the durability of DPP-4 inhibitor. DPP-4 inhibitor exerted beneficial effect for 12 months in some subjects whereas it did not at all in other subjects. Therefore, we divided the subjects into the effective group (*n* = 147) or ineffective group (*n* = 65) and examined the alteration of glycemic control in each group. We regarded the subjects as the effective group, when there was no change or a decrease of HbA1c level from baseline to 12 months in case of baseline HbA1c < 7% or when there was a decrease of HbA1c level from baseline to 12 months in case of baseline HbA1c ≥ 7%. As the results, HbA1c level at every point after dosing in the effective group was significantly lower compared to baseline HbA1c (overall trend; *p* < 0.0001) (Figure 1(b)). Conversely, HbA1c level after administrating in the ineffective group was significantly higher compared to baseline HbA1c (overall trend; *p* < 0.01). The value of HbA1c in the effective group was significantly lower than that in the ineffective group 12 months after starting the therapy (*p* < 0.0001) (Figure 1(b)).

To elucidate the factors related with the efficacy of DPP-4 inhibitors in subjects with type 2 diabetes, we analyzed the difference of clinical background between the effective group and ineffective group. Age (*p* < 0.05) and baseline HbA1c (*p* < 0.01) were significantly higher in the effective group than those in the ineffective group (Table 1). Although the baseline BMI was comparable between the groups, the value of body weight change tended to be lower in the effective group compared to that in the ineffective group (Table 1). Both the average of total cholesterol and average of triglyceride for over 1 year also tended to be lower in the effective group than those in the ineffective group (Table 1).

To elucidate the independent factors determining the efficacy of DPP-4 inhibitor in all participants with type 2 diabetes, we performed a multivariate regression analysis. Since there was some difference between the effective group and ineffective group in baseline HbA1c, body weight change, average total cholesterol, and average triglyceride (Table 1), we included baseline HbA1c, body weight change, average total cholesterol, and average triglyceride as independent variables. In addition, to avoid the possible influence of age or gender, we included age and gender as independent variables as well. As shown in Table 2, even after adjustment with age and gender, average triglyceride and basal HbA1c were independent factors contributing to the efficacy of DPP-4 inhibitor in subjects with type 2 diabetes. These data suggest that triglyceride management is very important to maintain the durability of DPP-4 inhibitor.

Next, we examined whether there were significant differences in the clinical background between the effective group and ineffective group in the presence or absence of obesity. In the nonobese group (BMI < 25 kg/m^2^) (*n* = 124), there was no difference between the effective group and ineffective group except for baseline HbA1c (*p* = 0.02; Table 3). In contrast, in the obese group (BMI ≥ 25 kg/m^2^) (*n* = 88), age was more advanced in the effective group compared with that in the ineffective group (*p* = 0.0008; Table 4). Conversely, average total cholesterol (*p* = 0.04), baseline triglyceride (*p* = 0.02), and average triglyceride (*p* = 0.005) were lower in the effective group than those in the ineffective group (Table 4).

To elucidate the independent factors determining the efficacy of DPP-4 inhibitor in the obese group with type 2 diabetes, we performed a multivariate regression analysis. Since there was difference between the effective group and ineffective group in baseline HbA1c, body weight change, average total cholesterol, and average triglyceride (Table 4), we included average total cholesterol and average triglyceride as independent variables. In addition, to avoid the possible influence of age or gender, we included age and gender as independent variables as well. As shown in Table 5, even after adjustment with age and gender, average triglyceride level was an independent factor contributing to the efficacy of DPP-4 inhibitor in the obese group with type 2 diabetes. These data suggest that triglyceride management is particularly important in obese subjects to maintain the durability of DPP-4 inhibitor.

## 4. Discussion

The final targets of diabetes treatment are extension of healthy life expectancy and preservation of the quality of life. It is necessary to keep favorable glycemic control in order to achieve these goals. A Diabetes Outcome Progression Trial (ADOPT) indicated that glucose-lowering effect by monotherapy (rosiglitazone, metformin or glyburide) was attenuated over the long term because of gradual decline of pancreatic *β*-cell function, although the efficacy of rosiglitazone was relatively preserved compared to that of other drugs [28, 29]. It has been shown that incretin-related drugs such as DPP-4 inhibitors preserve *β*-cell function and keep superior glucose-lowering effect compared with sulfonylurea [30–32]. Based on these backgrounds, incretin-related drugs have become a central agent in insulin secretagogues. Although recent studies reported that inadequate compliance with diet/exercise therapy and weight gain may be associated with an increase of HbA1c over time during treatment with DPP-4 inhibitor [26, 27], it is poorly understood about which factors are closely associated with the durability of glucose-lowering effect by DPP-4 inhibitor.

In this study, the effective group showed more advanced age, higher baseline HbA1c, tendency of smaller weight gain, lower average total cholesterol, and lower average triglyceride compared with the ineffective group. Multivariate regression analysis after adjustment with age and gender demonstrated that average triglyceride and basal HbA1c were independent factors determining the durability of DPP-4 inhibitor in subjects with type 2 diabetes. Furthermore, multivariate regression analysis after adjustment with age and gender in only obese subjects demonstrated that only average triglyceride level was an independent factor determining the durability of DPP-4 inhibitor. To the best of our knowledge, the present study is the first report which revealed the importance of lipid control in order to maintain the efficacy of DPP-4 inhibitor especially in the obese group with type 2 diabetes. The present study showed that when the value of average triglyceride was decreased by 20, 50, and 100 mg/dl, odds ratios were 0.86, 0.69, and 0.48 in all cases. Similarly, when the value of average triglyceride was decreased by 20, 50, and 100 mg/dl, odds ratios were 0.86, 0.68, and 0.46 in obese subjects. These data also clearly suggest the importance of triglyceride management for the durability of DPP-4 inhibitor in subjects with type 2 diabetes.

Kang et al. showed that GLP-1 receptor mRNA expression and GLP-1-stimulated insulin secretion were reduced in palmitate-treated rodent insulinoma cell lines and mouse islets [22], and GLP-1 receptor mRNA expression was restored in isolated islet in *db*/*db* mice treated with bezafibrate for 2 weeks compared to untreated *db*/*db* mice [23]. Moreover, lipid lowering by bezafibrate enhanced the efficacy of DPP-4 inhibitor des-fluoro-sitagliptin to improve glucose tolerance in *db*/*db* mice with amelioration of *β*-cell function [23]. Furthermore, previous reports revealed that fatty acid-mediated downregulation of connexin36 expression [24, 33], which consolidates the physiological and biochemical connectivity of neighboring *β*-cells [34] and disrupts incretin-regulated human *β*-cell connectivity [24]. Abnormality of lipid metabolism might decrease incretin effect at least partly through these mechanisms in *β*-cells. In fact, obesity is an independently predicted factor of decreased incretin action, and integration of obesity and hyperglycemia additively impair insulin secretion by incretin [21, 22]. Furthermore, previous articles showed that the short-term [35–37] and long-term [26] glucose-lowering effects by DPP-4 inhibitor were less in obese people with type 2 diabetes. In the present study as well as the past reports [26, 27], body weight gain tended to decrease the glucose-lowering effect over time during treatment with DPP-4 inhibitor. But multivariate regression analysis, which included both body weight change and average triglyceride as independent variables, demonstrated that average triglyceride was an independent factor determining the durability of DPP-4 inhibitor in subjects with type 2 diabetes. Moreover, there was a substantial difference between obese and nonobese individuals in the influence of triglyceride on glucose-lowering effect by DPP-4 inhibitor. These results indicate the importance of triglyceride management during treatment with DPP-4 inhibitor in obese subjects rather than nonobese subjects with type 2 diabetes.

There are some limitations to interpret the results of this study. First, DPP-4 inhibitor improves glycemic control through the action on pancreatic islets by increment of incretin hormone levels in the physiological range. Therefore, although we speculate that hypertriglyceridemia might impair the incretin action in the pancreatic islets, we could not examine the relationship between triglyceride level and islet function. Second, we could not clarify the relation between the efficacy of DPP-4 inhibitor and that of TG-lowering drugs (e.g., fibrate and eicosapentaenoic acid), because there were only a few people (18/212; 8.5%) who took these drugs. Third, the previous reports showed that DPP-4 inhibitor is effective in patients with type 2 diabetes with high serum eicosapentaenoic acid concentrations [36, 38]. Although we should conduct the analysis including the information related with dietary habits, we could not analyze this point owing to a retrospective study. Fourth, because blood samples were collected at various time points when the participants visited the hospital, the threshold of triglyceride level for the durability of efficacy of DPP-4 inhibitor remains uncertain. Further study would be necessary to demonstrate these points.

Taken together, the data in this study suggest that inadequate triglyceride management worsens the durability of DPP-4 inhibitor in subjects, particularly in obese subjects, with type 2 diabetes mellitus. Therefore, in order to maintain glucose-lowering effect by DPP-4 inhibitor for a long time, it is necessary to keep strict triglyceride management especially in obese subjects with type 2 diabetes.

## Figures and Tables

**Table 1:** Comparison of clinical background between the effective group and ineffective group in all participants with type 2 diabetes.

Clinical parameter	Effective group	Ineffective group	*p* value
Number	147	65	
Gender (men/women); *n* (%)	90 (61)/57 (39)	36 (55)/29 (45)	N.S.
Age (year)	65.8 ± 10.5	61.1 ± 14.3	<0.05
BMI (kg/m^2^)	25.1 ± 5.0	25.1 ± 5.5	N.S.
Duration of diabetes (year)	15.8 ± 9.6	13.5 ± 7.9	N.S.
Family history of diabetes (%)	60	49	N.S.
Baseline HbA1c (%) (mmol/mol)	7.8 ± 1.2	7.4 ± 1.0	<0.01
	62 ± 13.1	57 ± 10.9	
Body weight change (kg)	0.5 ± 4.1	0.8 ± 3.5	0.06
Total cholesterol (mg/dl)			
Baseline	182 ± 33	183 ± 35	N.S.
Average over 1 year	176 ± 27	184 ± 31	0.08
Triglyceride (mg/dl)			
Baseline	120 ± 65	145 ± 106	N.S.
Average over 1 year	120 ± 50	154 ± 101	0.09
HDL cholesterol (mg/dl)			
Baseline	56 ± 14	53 ± 15	N.S.
Average over 1 year	55 ± 13	53 ± 14	N.S.
LDL cholesterol (mg/dl)			
Baseline	98 ± 27	101 ± 27	N.S.
Average over 1 year	95 ± 19	100 ± 23	N.S.

BMI: body mass index; LDL: low-density lipoprotein; HDL: high-density lipoprotein; N.S.: not significant. Data are described as mean ± SD.

**Table 2:** Multiple logistic regression analysis of factors related with efficacy of DPP-4 inhibitor in all participants with type 2 diabetes.

Clinical parameter	Odds ratio	95% CI	*p* value
Baseline HbA1c	1.69	[1.217, 2.472]	0.004
Average triglyceride	0.99	[0.990, 0.999]	0.024
Age	1.03	[1.000, 1.064]	0.048
Average total cholesterol	0.99	[0.979, 1.003]	0.145
Gender	1.28	[0.655, 2.508]	0.466
Body weight change	1.01	[0.934, 1.104]	0.731

Dependent variable: effective group. Gender: 0, female; 1, male. Odds ratio: per 1-increment increase in variable. CI: confidence interval.

**Table 3:** Comparison of clinical background between the effective group and ineffective group in nonobese subjects with type 2 diabetes (BMI < 25 kg/m^2^).

Clinical parameter	Effective group	Ineffective group	*p* value
Number	89	35	
Gender (men/women); *n* (%)	56 (63)/33 (37)	18 (51)/17 (49)	N.S.
Age (year)	68.9 ± 9.4	69.0 ± 10.2	N.S.
BMI (kg/m^2^)	22.1 ± 1.9	21.4 ± 2.6	N.S.
Duration of diabetes (year)	17.8 ± 9.9	15.2 ± 8.4	N.S.
Family history of diabetes (%)	66	44	N.S.
Baseline HbA1c (%) (mmol/mol)	7.7 ± 1.0	7.1 ± 0.7	0.0003
	61 ± 10.9	54 ± 7.7	
Body weight change (kg)	0.5 ± 2.4	1.0 ± 2.7	N.S.
Total cholesterol (mg/dl)			
Baseline	180 ± 32	173 ± 32	N.S.
Average over 1 year	175 ± 29	179 ± 31	N.S.
Triglyceride (mg/dl)			
Baseline	109 ± 59	96 ± 44	N.S.
Average over 1 year	108 ± 48	108 ± 46	N.S.
HDL cholesterol (mg/dl)			
Baseline	58 ± 15	56 ± 17	N.S.
Average over 1 year	57 ± 14	56 ± 16	N.S.
LDL cholesterol (mg/dl)			
Baseline	95 ± 25	93 ± 21	N.S.
Average over 1 year	93 ± 20	95 ± 22	N.S.

BMI: body mass index; HDL: high-density lipoprotein; LDL: low-density lipoprotein; N.S.: not significant. Data are described as mean ± SD.

**Table 4:** Comparison of clinical background between the effective group and ineffective group in obese subjects with type 2 diabetes (BMI ≥ 25 kg/m^2^).

Clinical parameter	Effective group	Ineffective group	*p* value
Number	58	30	
Gender (men/women); *n* (%)	34 (59)/24 (41)	18 (60)/12 (40)	N.S.
Age (year)	60.9 ± 10.5	51.8 ± 12.8	0.0008
BMI (kg/m^2^)	29.6 ± 4.9	29.4 ± 4.8	N.S.
Duration of diabetes (year)	12.7 ± 8.3	11.3 ± 6.9	N.S.
Family history of diabetes (%)	51	55	N.S.
Baseline HbA1c (%) (mmol/mol)	8.0 ± 1.6	7.8 ± 1.2	N.S.
	64 ± 17.5	62 ± 13.1	
Body weight change (kg)	0.5 ± 5.7	0.7 ± 4.3	N.S.
Total cholesterol (mg/dl)			
Baseline	185 ± 35	196 ± 35	N.S.
Average over 1 year	177 ± 23	190 ± 29	0.04
Triglyceride (mg/dl)			
Baseline	139 ± 70	202 ± 127	0.02
Average over 1 year	137 ± 49	209 ± 120	0.005
HDL cholesterol (mg/dl)			
Baseline	53 ± 12	50 ± 12	N.S.
Average over 1 year	52 ± 11	50 ± 11	N.S.
LDL cholesterol (mg/dl)			
Baseline	103 ± 31	110 ± 30	N.S.
Average over 1 year	98 ± 19	106 ± 24	N.S.

BMI: body mass index; LDL: low-density lipoprotein; HDL: high-density lipoprotein; N.S.: not significant. Data are described as mean ± SD.

**Table 5:** Multiple logistic regression analysis of factors related with efficacy of DPP-4 inhibitor in obese subjects with type 2 diabetes (BMI ≥ 25 kg/m^2^).

Clinical parameter	Odds ratio	95% CI	*p* value
Age	1.06	[1.010, 1.107]	0.019
Average triglyceride	0.99	[0.984, 0.999]	0.033
Average total cholesterol	0.98	[0.962, 1.005]	0.152
Gender	1.26	[0.438, 3.688]	0.665

Odds ratio: per 1-increment increase in variable. CI: confidence interval.
